# Integrating Phosphate Enhances Biomineralization Effect of Methacrylate Cement in Vital Pulp Treatment with Improved Human Dental Pulp Stem Cells Stimulation

**DOI:** 10.1002/adhm.202402397

**Published:** 2024-10-04

**Authors:** Jeong‐Hyun Ryu, Utkarsh Mangal, Jae‐Sung Kwon, Ji‐Young Seo, Seong‐Yun Byun, Young‐Hee Lee, Sungil Jang, Geelsu Hwang, Hyemin Ku, Yooseok Shin, Dohyun Kim, Sung‐Hwan Choi

**Affiliations:** ^1^ Department of Orthodontics Institute of Craniofacial Deformity Yonsei University College of Dentistry Seoul 03722 Republic of Korea; ^2^ BK21 FOUR Project Yonsei University College of Dentistry 50–1 Yonsei‐ro, Seodaemun‐gu Seoul 03722 Republic of Korea; ^3^ Department and Research Institute of Dental Biomaterials and Bioengineering Yonsei University College of Dentistry 50–1 Yonsei‐ro, Seodaemun‐gu Seoul 03722 Republic of Korea; ^4^ Department of Oral Biochemistry Institute of Oral Bioscience School of Dentistry Jeonbuk National University Jeonju‐si 54907 Republic of Korea; ^5^ Department of Preventive and Restorative Sciences School of Dental Medicine University of Pennsylvania Philadelphia PA 19104 USA; ^6^ Center for Innovation & Precision Dentistry School of Dental Medicine School of Engineering and Applied Sciences University of Pennsylvania Philadelphia PA 19104 USA; ^7^ Department of Conservative Dentistry and Oral Science Research Center Yonsei University College of Dentistry 50–1 Yonsei‐ro, Seodaemun‐gu Seoul 03722 Republic of Korea

**Keywords:** dentin, DPSCs, methacrylate cements, odontogenesis, phosphate, pulp, vital pulp treatment

## Abstract

Vital pulp treatment (VPT) is crucial for preserving the health and function of the tooth in cases where the pulp tissue remains vital despite exposure. Various materials are introduced for this purpose. However, challenges such as low strength, high solubility, and tooth discoloration persist. Methylmethacrylate‐based cement (MC) offers excellent sealing ability, feasibility, and mechanical properties, making it a promising alternative for VPT. Phosphate‐based glass (PBG) has the potential to promote hard tissue regeneration by releasing key inducers, phosphorus (P) and calcium (Ca), for reparative odontogenesis. This study investigates PBG‐integrated MC (PIMC) by characterizing its properties, assessing human dental pulp stem cell activity related to initial inflammatory adaptation and odontogenic differentiation, and evaluating hard tissue formation using an in vivo dog pulpotomy model. Results indicate that a 5% PBG‐integrated MC (5PIMC) maintains the physicochemical properties of MC. Furthermore, 5PIMC demonstrates cytocompatibility, excellent expression of osteo/odontogenic markers, and resistance to inflammatory markers, significantly outperforming MC. Enhanced hard tissue formation is observed in the dental pulp of mongrel dog teeth treated with 5PIMC. These findings suggest that 5PIMC could be an optimal and suitable material for reparative odontogenesis through VPT.

## Graphical abstract

The integration of phosphate‐based glass into methylmethacrylate‐based cement (PIMC) presents promising characteristics for vital pulp treatment. PIMC demonstrates persistent strength and stability in wet conditions, effectively controls initial inflammation, and stimulates human dental pulp stem cells. Overall, PIMC supports mineralized tissue repair at the dentin‐pulp interface, making it an excellent candidate as a bioactive material for vital pulp therapy.

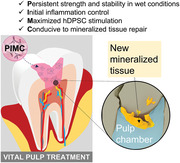

## 1 Introduction

The dental pulp is highly vascularized and richly supplied with nerves that connect to periodontal tissues.^[^ ^1^ ^]^ From a functional anatomy perspective, the dentin‐pulp complex plays a crucial role in supporting the tooth against masticatory and occlusal forces.^[^ ^2^ ^]^ However, damage in dental hard tissues such as deep dental caries and fractures can result in dental pulp exposure, which causes inflammatory responses and ultimately leads to necrosis of dental pulp tissues.^[^ ^3^ ^]^

Vital pulp treatment (VPT) aims to preserve the vitality and function of the dental pulp after injury resulting from dental trauma, caries, or iatrogenic reasons. This treatment involves indirect or direct pulp capping and pulpotomy by applying VPT materials such as calcium hydroxide, glass ionomer cements, resin‐based materials, and calcium silicate cements directly over affected tissues.^[^ ^4^ ^]^ For successful VPT, the material should provide a tight seal against bacterial leakage, stimulate healing of the dental pulp tissue, and facilitate reparative odontogenesis.^[^ ^5^ ^]^ Calcium hydroxide and calcium silicate cements have been preferably used in VPT due to their antibacterial effects, high alkalinity, and ability to stimulate reparative dentin formation.^[^ ^6^ ^]^ However, they face issues such as poor adhesion, solubility, tooth discoloration, and cytotoxicity.^[^ ^7^ ^]^

Methyl methacrylate‐based cement (MC) belongs to the polymer based on acrylic resins, which is a widely used bone cement approved by the US Food and Drug Administration.^[^ ^8^ ^]^ MC has been commonly used in hard tissue applications due to its high mechanical properties, sealing ability, feasibility, and biocompatibility in the biomedical field for several decades.^[^ ^9^ ^]^ For these advantages, MC is a promising new material that could potentially possess the required properties of endodontic materials.^[^ ^10^ ^]^ In particular, MC has been widely utilized for anchoring prostheses to living bone, reflecting significant changes in its utilization trend. Previous studies have demonstrated that MC has the lowest level of microleakage attributed to its excellent sealing ability and good adaptation to dentinal walls.^[^ ^9^, ^11^ ^]^ Hence, the characteristics of MC make it a suitable candidate for use as an endodontic material.

To enhance the suitability of MC as an endodontic biomaterial, researchers have incorporated various ion‐rich bioactive inorganic additives. One notable additive is phosphate‐based glass (PBG), which possesses unique bioresorbable properties and promotes hard tissue regeneration by releasing beneficial ions such as phosphorus (P) and calcium (Ca) under physiological conditions.^[^ ^12^ ^]^ PBG composed of tetrahedral phosphate unit linkages can release bioactive ions. Specifically, inorganic phosphate plays a critical role in dentin sialophosphoprotein (DSPP) and dentin matrix protein‐1 (DMP1) that contribute to the formation of the extracellular matrix (ECM) in dentin‐like mineralized tissues.^[^ ^13^ ^]^Although the hard tissue regeneration effect of PBG through P and Ca release has been well established, translating this into a clinically relevant endodontic material remains challenging due to concerns over maintaining bioactive effects while retaining optimal physical characteristics. Previous studies exploring the role of additives in methacrylate networks have shown that excessive additives such as bioceramics or inorganic compounds can disrupt the physicochemical and mechanical properties of the material.^[^ ^14^ ^]^

**Figure 1:**
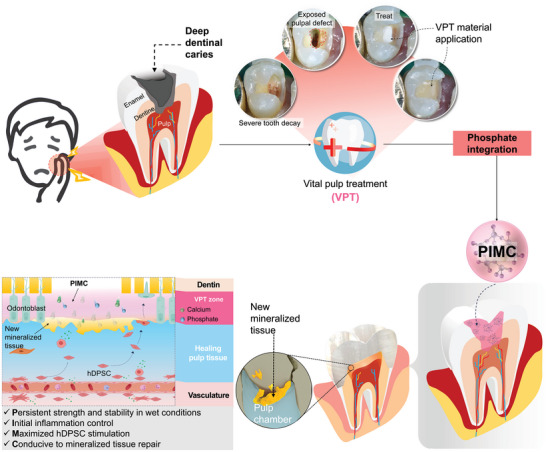
Schematic of phosphate‐based glass‐integrated methacrylate‐based cement (PIMC) for vital pulp treatment (VPT). PIMC supports the bioactive cellular activity of human dental pulp stem cells (hDPSC), promoting the formation of odontogenic‐like hard tissue and enhancing the regenerative response to pulp exposure.

To address these challenges, this study aimed to optimize the PBG‐integrated MC resin (PIMC) matrix to balance its bioactive and physical properties. We hypothesized that a composite material with desirable mechanical properties of MC and enhanced bioactivity for effective VPT could be obtained by carefully controlling the amount of PBG. To this end, we investigated the physicochemical and mechanical properties of PIMC with varying concentrations of PBG to determine the optimal formulation. Focusing on dentin biomineralization for VPT, we characterized the inflammatory and odontogenic expression of human Dental Pulp Stem Cells (hDPSC) under optimized PIMC treatment. Finally, to develop a clinically viable endodontic material, we evaluated the formation of reparative dentin‐like hard tissue following vital pulp treatment in vivo using a dog tooth pulpotomy model. This comprehensive approach ensures that the developed material can effectively promote hard tissue formation without compromising structural integrity (**Figure** **1**).

## 2 Results and Discussion

### 2.1 Characterization of MC and PIMC

**Figure 2:**
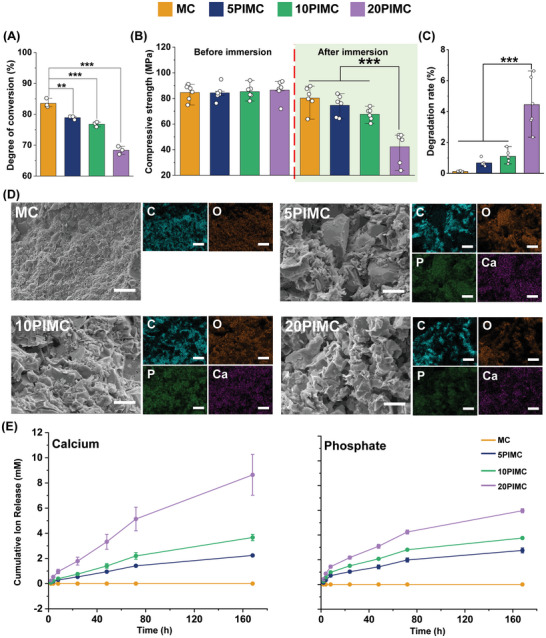
Characterization of PIMC groups. A) Degree of conversion (DC) of PIMC evaluated by attenuated total reflection Fourier transform infrared spectroscopy (ATR‐FTIR) (*n* = 3; **p < 0.01, ***p < 0.001). B) Compressive strength of PIMC groups before and after immersion in distilled water for 7 days (*n* = 6; ***p < 0.001). C) Degradation rate of PIMC groups after immersed in distilled water for 7 days (*n* = 6; ***p < 0.001). D) Morphology and elemental analysis of PIMC groups detected by Ca and P as composed of PBG. E) The ionic release profile of P and Ca from PIMC groups for 168 h (*n* = 3). MC, methacrylate‐based cement; PBG, phosphate‐based glass; PIMC, PBG‐integrated MC; Ca, calcium; P, phosphorous.

To comprehensively understand the sensitivity of PBG integration, PIMC groups were compared for essential properties determining the applicability. Considering the inadvertent engagement of additive particles in the resin matrix during free radical polymerization, degree of conversion (DC%) was compared.^[^ ^15^ ^]^ Accounting for a significant reduction in the dough time following an increase in viscosity with PBG concentration (Figure S1, Supporting Information), samples were analyzed following an incubation period (24 h) after setting.^[^ ^16^ ^]^ PIMC groups showed a progressive reduction in DC% with an increase in PBG loading. 5PIMC and 10PIMC groups had relatively fewer reductions of 5.5% and 8.1% than the 20PIMC which showed a marked reduction of ≈18.1% compared to the MC group (**Figure** **2**A). These observations could also be attributed to the lower L/S that can restrict the availability of monomers during polymerization.

It is desirable for VPT materials to exhibit mechanical strength capable of withstanding masticatory functional loads while also buffering impact on underlying affected dentin or pulp. All PIMC groups demonstrated favorable compressive strength without adverse effects from PBG addition (Figure 2B, before immersion). However, the inclusion of PBG in the resin matrix can induce hydrophilicity, leading to higher retention of water molecules within the matrix.^[^ ^17^ ^]^ This was corroborated by water contact angle analysis results, which showed a reduction of up to 34.6% in contact angle in the 20PIMC group (Figure S2, Supporting Information). Further investigation of the hydrophilicity effect on PIMC revealed a significant (*p* < 0.001) loss in the compressive strength after 7‐day PBS immersion (Figure 2B, after immersion). Rapid degradation was observed in the 20 PIMC group (Figure 2C). In contrast, the 5PIMC group was relatively stable, retaining a compressive strength of 79.2 MPa with less than 1% rate of degradation (Table S3, Supporting Information). Additional SEM investigation (Figure 2D) further confirmed that the PBG was homogenized within the PIMC, with this effect primarily driven by a hydrolytic process. The changes following immersion were further validated by comparing the surface morphologies observed after immersion (Figure S3, Supporting Information).

Results of analyzing ion release from PIMC, a predictable trend showing incremental concentration of Ca and P with an increase in PBG incorporation was found (Figure 2E). However, the increase with 10PIMC was statistically higher than that with 5PIMC (Tables S4 and S5, Supporting Information). Although Ca and P ions are essential ions for eliciting a bioactive response from hDPSC, compromised strength and stability are undesirable for an effective VPT.

In summary, 5PIMC demonstrated a highly stable configuration with effective ion release following homogeneous incorporation of PBG. Consequently, for subsequent biological analyses, 5PIMC was compared against MC.

### 2.2 Cytocompatibility and Early Inflammatory Response of hDPSC

**Figure 3:**
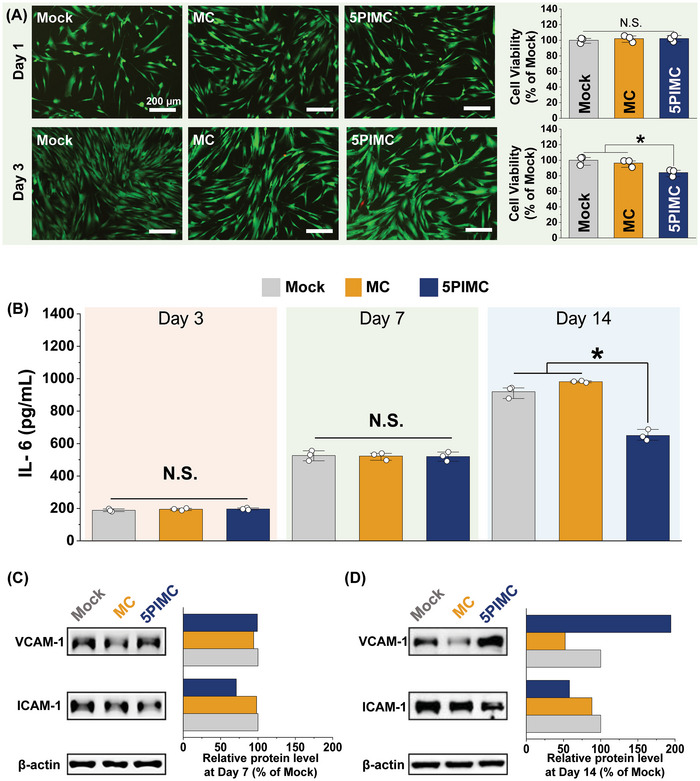
Cytotoxicity and inflammatory response of hDPSCs. A) Cytocompatibility of PIMC by live/dead (green/red) cell staining and WST‐1 assay after 1 day and 3 days (*n* = 3; *p < 0.05; NS, no significance). Expression of pro‐inflammatory cytokine for odontogenic differentiation from hDPSC is shown. B) Detection of IL‐6 from cultured supernatant using ELISA at 3, 7, and 14 days (*n* = 3; *p < 0.05; NS, no significance). C) Relative protein expression of ICAM‐1 and VCAM‐1 in odontogenesis from differentiation of hDPSC after 14 days of treatment. hDPSC, human dental pulp stem cell; ICAM‐1, intracellular adhesion molecule‐1; VCAM‐1, vascular cell adhesion molecule‐1; MC, methacrylate‐based cement; PBG, phosphate‐based glass; PIMC, PBG‐integrated MC; hDPSC, human dental pulp stem cell.

Post‐treatment sensitivity with dental cements has been reported under restorations for deep dentinal caries.^[^ ^18^ ^]^ This sensitivity can be attributed to two primary factors: the exothermic reaction, which causes a rapid increase in the temperature of the surrounding tissues, and the elution of cement constituents. Our preliminary findings indicate that the maximum temperature reached, 36.87 °C, remains safely below the potentially harmful threshold of 42.5 °C, as defined by ISO 5833:2002.^[^ ^19^ ^]^ Particularly in VPT, the risk of an aggravated response is high due to proximity to the underlying pulp tissue. Therefore, to investigate the biological effects of 5PIMC eluates in VPT, we examined their cytocompatibility and early inflammatory cytokine expression in hDPSCs. Co‐incubated cells on day 1 presented a similar response for all groups. Following a 3‐day co‐culture, all 5PIMC‐treated samples retained cell viability above 85% at the original concentration (**Figure** **3**A). In particular, the cell viability of 5PIMC was lower than that of MC due to the entered differentiation phase of cellular behavior simulated by Ca and P concentration in agreement with previous literature.^[^ ^20^ ^]^ Cytotoxicity assays with varying Ca and P concentrations in cements also showed similar findings, highlighting that higher P concentrations could result in better cell viability, as observed after culture for up to 5 days.^[^ ^21^ ^]^

For early inflammatory response, we assayed changes in IL‐6 level from cell culture supernatant after co‐culture with eluates. IL‐6 is an important pro‐inflammatory cytokine involved not only in the activation of the immune system but also in regenerative processes.^[^ ^22^ ^]^ In differentiating odontoblast‐like cells, IL‐6 levels are down‐regulated due to anti‐inflammatory response during tissue regeneration.^[^ ^23^ ^]^ After culturing for 3 and 7 days, IL‐6 levels in the MC and 5PIMC groups were not significantly different from those in the mock group. By 14 days of culture, the 5PIMC group demonstrated a favorable outcome with an approximate 38% reduction in IL‐6 level compared to the MC group (Figure 3B). High levels of IL‐6 levels and progressively increasing IL‐6 levels can affect the adhesion to dentin and the biomineralization potential of hDPSC.^[^ ^24^ ^]^ The observed reduction in IL‐6 level after 14 days validated a stimulatory bioeffect of 5PIMC, with IL‐6 levels markedly below those of cells isolated from inflamed pulp tissue, indicating a favorable environment for regeneration.^[^ ^24^ ^]^

Concurrently, we investigated protein levels of inflammatory molecules such as ICAM‐1 and VCAM‐1 in subsequent experiments after cells were cultured for 7 days (Figure 3C) and 14 days (Figure 3D). ICAM‐1 and VCAM‐1 are key cell adhesion molecules that play important roles in the recruitment and transmigration of inflammatory cells in the immune system. The level of ICAM‐1 expression was substantially lower in 5PIMC‐treated cells than in MC‐treated cells (≈28% of the MC group). After 14 days, the 5PIMC group presented a reduced ICAM‐1 protein level by ≈34% compared to the MC group. For hDPSC, the reduction of ICAM‐1 expression in the microenvironment could promote odontogenic activity, aiding healing at the crucial interface of the dentin and pulp.^[^ ^25^ ^]^

Typically, VCAM‐1 mediates the proliferation and differentiation of mesenchymal stem cells.^[^ ^26^ ^]^ The expression of VCAM‐1 regulates the osteogenic differentiation of hDPSCs through PI3K/Akt pathways such that cells with high expression of VCAM‐1 in an undifferentiated state are more likely to differentiate.^[^ ^27^ ^]^ While VCAM‐1 expression from 5PIMC after 7 days of treatment showed a mild increase, it showed a significantly higher expression (37.1‐fold) after 14 days of treatment. These results confirmed that 5PIMC could enhance bioactive stimulation to promote odontogenic activity in hDPSCs.

### 2.3 PIMC Stimulates Osteogenic/Odontogenic Differentiation Potential of hDPSC

**Figure 4:**
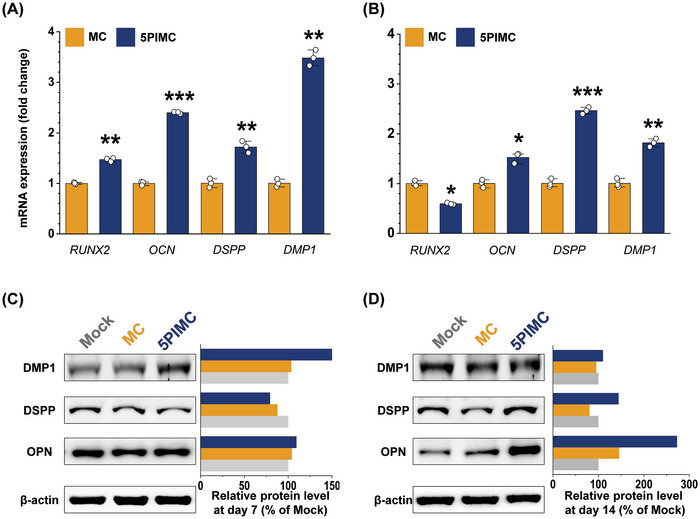
Odontogenic expression assay of DPSC differentiation. A,B) Relative mRNA expression levels normalized against the MC group at (A) day‐7 and (B) day‐14 after treatment (*n* = 3; *p < 0.05; **p < 0.01; ***p < 0.001). C,D) Relative protein level expression from PIMC and MC groups compared to the mock group at (C) day‐7 and (D) day‐14 after treatment. RUNX2, runt‐related transcription factor 2; OCN, osteocalcin; OPN, osteopontin; DSPP, dentin sialopphosphoprotein; DMP1, dentin matrix protein 1. MC, methacrylate‐based cement; PBG, phosphate‐based glass; PIMC, PBG‐integrated MC.

To explore the effects of key constituent ions, Ca and P, on osteogenic/odontogenic stimulation potential, we evaluated 5PIMC‐treated hDPSCs after 7 and 14 days of culture. Assessing typical odontogenic differentiation markers from hDPSCs revealed an enhanced early differentiation response at day 7 with an increase of ≈48% for RUNX2, 139% for OCN, 71% for DSPP, and 247% for DMP1 following 5PIMC treatment as compared to the MC group (**Figure** **4**A). Furthermore, at day 14, hDPSCs expression after 5PIMC treatment showed an augmented expression by ≈52% for OCN, 146% for DSPP, and 81% for DMP1 as compared with the MC group (Figure 4B).

For the maturation of odontoblast‐like cells derived from DPSC, the secreted ECM network composed by DPSC was attributed to facilitated odontogenic activity by augmentation of key odontogenic markers.^[^ ^28^ ^]^ The assay conducted at 7 days post‐treatment with 5PIMC showed strong expression of DMP1 transcript, which was more than 30% higher than MC‐treated cells (Figure 4C). Notably, when groups were compared after 14 days of culture following 5PIMC treatment, significantly dominant transcript expression was observed for DMP1 (10%), DSPP (44%), and OPN (173%) (Figure 4D). Typically, during matrix formation, DMP‐1 upregulation precedes cellular commitment to differentiation. Given the end goal of VPT for reparative odontogenesis, DMP‐1 plays a critical role in establishing the initial matrix.^[^ ^29^ ^]^

Subsequent matrix maturation in reparative odontogenesis is supported by OPN upregulation. OPN is recognized in the dentin and at the boundary between the tertiary dentin and preexisting dentin.^[^ ^30^ ^]^ After cavity preparation during VPT, immature odontoblast‐like cells also express OPN.^[^ ^31^ ^]^ This upregulation of OPN following osteo/odontogenic induction of hDPSCs aids in the maturation process. In agreement with the above, our results showed increased activity indicative of matrix formation on day 7. By day 14, strong expression of DSPP, a gene highly selective to odontoblasts,^[^ ^32^ ^]^ validated the successful osteogenic/odontogenic induction of 5PIMC‐treated cells accompanied by high OPN expression.

Given the stimulatory osteogenic effect on mesenchymal‐origin stem cells, we additionally characterized the response from pre‐osteoblast cells (MC3T3‐E1) and observed that the effect extended to these cells as well, enhancing their differentiation and activity. Cells presented good cell viability without a marked loss of morphology within 24 h and after 3 days of culture following treatment (Figure S4A, Supporting Information). Previous studies showed that acrylic resin with 5 wt.% additives could facilitate the growth of pre‐osteoblasts without inducing cell apoptosis.^[^ ^9^, ^14^ ^]^ Similar to earlier work, a favorable cellular proliferation response was confirmed on day 3.^[^ ^14^, ^33^ ^]^ Subsequently, after 7‐day culture, pre‐osteoblastic cells showed a significant increase in mRNA expression of early osteogenic markers, with all markers showing elevated levels in the 5PIMC group. Specifically, a more than 2‐fold increase was observed in OPN and a 4‐fold increase was observed for OCN and BSP levels compared to MC‐treated cells (Figure S4B, Supporting Information). These results further confirmed that PBG could enhance osteogenic differentiation and augment cellular response.^[^ ^34^ ^]^

### 2.4 Effect of PIMC on In Vivo Hard Tissue Regeneration in Dental Pulp

**Figure 5:**
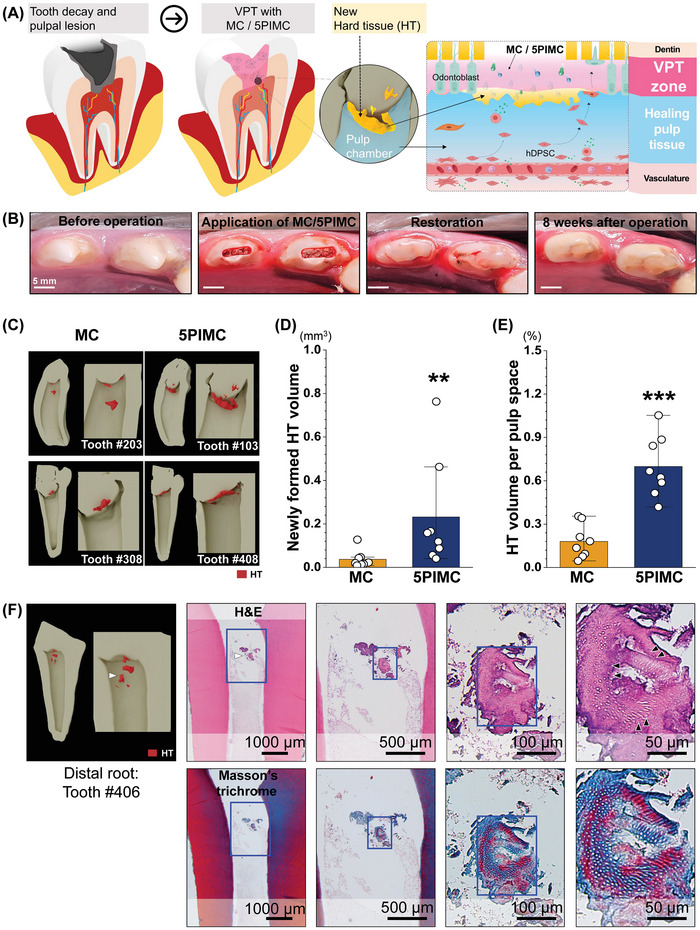
Effect of PIMC on hard tissue regeneration in dental pulp. A) Schematic representative of PIMC application for vital pulp treatment and hard tissue regeneration. B) Clinical photographs and radiographs for operative procedures on dog teeth. Scale: 5 mm. C) Reconstruction images of newly formed HT formation in the pulp space (red‐colored area) analyzed by µ‐CT. D,E) Volume of newly formed hard tissue and the volume ratio of hard tissue to soft tissue measured by µ‐CT (*n* = 8; *p < 0.05; ***p < 0.001). F) Histological images of newly formed HT in the pulp space (white arrowheads) demonstrating tubular structures (black arrowheads) resemble physiologic dentin in a tooth of the 5PIMC group. MC, methacrylate‐based cement; PBG, phosphate‐based glass; PIMC, PBG‐integrated MC; HT, hard tissue; µ‐CT, micro‐computed tomography.

VPTs are typically indicated for teeth that have a pulp exposure caused by trauma or deep caries, where the exposed pulp is still vital or inflamed with the ability to heal.^[^ ^35^ ^]^ The main functions of the pulp include the formation of new dentin.^[^ ^36^ ^]^ The pulp‐dentin complex regulates normal tissue architecture at the pulp periphery and repairs damaged dentin by new hard tissue formation. We found that ionic P and Ca released from the 5PIMC group affected the newly formed hard tissue due to stimulation of odontogenic activity from dental pulp stem cells as pulp capping materials (**Figure** **5**A).

To evaluate the in vivo response of dental pulp to 5PIMC compared with the MC, we applied materials at the orifice of each root canal in contact with the remaining pulp tissue after partial removal of the coronal pulp tissue from the tooth (Figure 5B; Figure S5, Supporting Information). A total of 16 roots were included, with 8 roots and contralateral 8 roots belonging to the MC group and the 5PIMC group, respectively. After 8 weeks, qualitative and quantitative analyses of hard tissue formation in the dental pulp were performed for the MC group and 5PIMC group. µ‐CT results depicted that the 5PIMC group enhanced hard tissue regeneration (red color area) compared to the contralateral tooth treated with MC (left) qualitatively (Figure 5C). In terms of quantification in µ‐CT analysis (Table S6, Supporting Information), the 5PIMC group showed significant newly formed hard tissues in pulp spaces against the MC group (Figure 5D). Especially, there was a significant (≈288%) augmentation of the volume ratio of hard tissue (HT) formation per pulp space (ST) in the 5PIMC group against the MC group (Figure 5E). These results suggested that 5PIMC could augment newly formed hard tissues and the ratio of hard tissue to soft tissue, in agreement with findings obtained from in vitro studies that P and Ca ions played a positive role in odontogenesis.

Ultimately, we validated that the 5PIMC group was biocompatible without any adverse effects. We also validated new hard tissue formation at the microscopic level by H&E staining through histological analysis after 8 weeks. Specifically, results demonstrated a tubular structure (black arrowheads) closely resembling physiologic dentin representing dentinal tubule structures composed of collagen (blue color) and mineralized region (red color) (Figure 5F).

The 5PIMC group exhibited favorable biosafety characterization by showing no inflammatory response such as necrosis or hypersensitivity reaction. This might be due to the use of biocompatible materials, as it was composed of MC and PBG based on previous literature.^[^ ^9^, ^12^ ^]^ Additionally, we observed that the newly formed hard tissue had regenerative properties due to orthophosphate released from PBG, which is known to play a crucial role in hard tissue formation.^[^ ^12b^ ^]^ A limitation of this study was the lack of long‐term observation of the dentin‐pulp complex in an in vivo model, particularly in comparison to the MC group. Future research should address this by designing detailed in vivo studies to investigate the long‐term response to 5PIMC and the immune response in a clinical environment, building upon the findings of this study. Although our primary objective was to explore the unique properties of 5PIMC, it is equally crucial to compare these findings with current commercial standards, such as mineral trioxide aggregate or Biodentine. The detailed characterization provided in this study offers a foundational understanding of 5PIMC's intrinsic properties, which can serve as a benchmark against these established standards in future research. Moreover, the findings contribute valuable scientific evidence through in‐depth biological characterization, laying the groundwork for continued research into the development of novel VPT materials.

## 3 Conclusion 

The current study demonstrated that the 5PIMC maintained physicochemical and mechanical properties. In vitro studies showed that 5PIMC promoted reparative odontogenesis by augmenting main odontogenic expression and resisting inflammatory markers, while VCAM‐1 contributed to inducing odontogenic activity. In vivo findings revealed that 5PIMC facilitated the regeneration of hard tissue compared to MC. Taken together, the results of the present study provide evidence that 5PIMC could be a promising material for VPT by promoting reparative odontogenesis.

## 4 Experimental Section

### Preparation of PIMC

Commercially available methacrylate‐based cement (MC; MIXMAX, Sewon Meditech, Bucheon‐si, Republic of Korea) was used as the base material. PBG was fabricated with the melt‐quenching method in accordance with a previous study.^[^ ^12b^ ^]^ Briefly, P_2_O_5_ (Sigma–Aldrich, St. Louis, MO, USA), CaCO_3_ (Sigma–Aldrich), and Na_2_CO_3_ (Sigma–Aldrich) powders were homogeneously mixed using a tubular shaker‐mixer (Model T2F, Glen Mills Inc. USA) for 60 min at 100 rpm (Table S1, Supporting Information). After that, the mixture of powders in an alumina crucible was melted at 1100 °C with a heating rate of 10 °C min^−1^ in an electric furnace (Lindberg, Asheville, NC, USA). The melted glass was quenched at 25 °C and then coarse ground on an alumina surface to produce PBG frit. The PBG powder was prepared using a planetary mono mill (Pulverisette‐7; Fritsch, Idar‐Oberstein, Germany). Subsequently, PBG powders were sieved through a 150‐micron mesh stainless‐steel sieve to obtain particles smaller than 150 µm. Four groups of PIMC were designed with four different mass ratios (0%, 5%, 10%, and 20%) of PBG to MC (**Table** **1**).

**Table 1:** Composition of methacrylate and PBG composite cement.

Code	Filler loading [wt%]	Methacrylate powder [g]^a)^	PBG [g]^b)^	Liquid [mL]^c)^	L^c^/S^a^ ^+^ ^b^ ^)^
MC	0	2	‐	1	0.5
5PIMC	5	2	0.1	1	0.48
10 PIMC	10	2	0.2	1	0.45
20 PIMC	20	2	0.4	1	0.42

^a)^ MC, Methacrylate‐based cement;

^b)^ PBG, Phosphate‐based glass;

^c)^ L/S, liquid to solid ratio.

### Dough Time

To handle characteristics in terms of dough time, the procedure was performed according to a previous study.^[^ ^37^ ^]^ Both the powder and liquid components of PIMC were mixed for 60 s. The mixture was then probed at 15‐s intervals using a nonpowdered, surgical‐gloved finger. Dough time was recorded when the mixture was cleanly separated from the gloved finger upon probing.

### Degree of Conversion

Specimens of PIMC groups were analyzed using Fourier‐transform infrared spectroscopy coupled with single‐reflection attenuated total reflectance (FTIR‐ATR) (Vertex 70, Bruker, Billerica, MA, USA) according to the previous study.^[^ ^14b^ ^]^ The DC% was calculated as the ratio between the aliphatic (C═C) peak at 1638 cm^−^¹ and the aromatic (C─C) peak at 1608 cm^−^¹ of methacrylate both before and after curing of specimens as described in previous studies.^[^ ^14^, ^38^ ^]^ The DC % was calculated using the following equation:

(1) $$DC\;\;\left(\%\right)=\;100\;\times \left(1-\frac{\left(\frac{1638}{1608}\right)Polymerized}{\left(\frac{1638}{1608}\right)Unpolymerized}\right)$$

### Surface Wettability

The surface wettability of PIMC specimens was measured using a static water contact angle goniometer (SmartDrop, Femtobiomed Inc., Seongnam, Republic of Korea) in accordance with the previous study.^[^ ^39^ ^]^ Each material was placed in a mold with a diameter of 10 mm and a height of 2 mm to form a disc‐shaped sample. A 3 µL drop of distilled water was placed on the surface of each material and the contact angle was measured to obtain results.

### Compressive Strength

Each material was placed in a mold with a diameter of 6 mm and a height of 12 mm to form cylindrical specimens for the compressive strength test in accordance with the previous study.^[^ ^9b^ ^]^ To determine the changes in compressive strength before and after immersion in the deionized water, the specimens were immersed in deionized water at a ratio of 10 mm^2^ mL^−1^ for 7 days following a previous study.^[^ ^9a^ ^]^ Subsequently, specimens were dried at 50 °C for 24 h in an oven. Compressive strength was calculated for both non‐immersed and immersed conditions. All tests were performed using an Instron 3366 universal testing machine (Instron, Norwood, MA, USA) at a crosshead speed of 20 mm min^−1^ until failure. The compressive strength was calculated using the following equation:

(2) $$\sigma \;=\frac{4P}{\pi d^{2}}\;$$

### Degradation Rate

Each material was prepared in a mold with a diameter of 6 mm and a height of 12 mm to form a cylindrical‐shaped sample. Specimens were immersed in deionized water at a ratio of 10 mm^2^ mL^−1^ for 7 days. After immersion, each specimen was dried at 50 °C for 24 h in an oven. The degradation rate of each specimen was then calculated using the following equation:

(3) $$\mathrm{WL}\left(\%\right)=\left(W_{0}-W_{t}\right)/W_{0}\times 100$$

where WL (%) was the degradation percentage, *W*_0_ was the initial weight, and *W*_t_ was the dried weight.

### Morphology and Element Analysis

To observe the morphology and conduct elemental analysis, each specimen was coated with platinum using a Cressington sputter coater 208HR (Cressington Scientific Instrument Ltd., UK) for 90 s at 20 mA. Specimens were then observed with a field emission scanning electron microscope (FE‐SEM, JEOL‐7800F, JEOL Ltd., Tokyo, Japan) operating at an accelerating voltage of 10 kV. Additionally, elemental mapping analysis was performed using an energy‐dispersive X‐ray spectrometer (EDS, JEOL Ltd) to determine the elemental compositions of each specimen. All specimens were prepared under similar conditions and analyzed without any additional treatment.

### Ion Release Profile

Following a previous study,^[^ ^9a^ ^]^ disc‐shaped specimens (diameter 10 mm, height 2 mm) were immersed in deionized water (pH 5.45 ± 0.3) at a ratio of 10 mm^2^ mL^−1^ for 168 h. The extraction conditions ensured that the pH levels during elution were maintained within the 4–10 range, which effectively minimizes H^+^ interference. At each selected time point, 2 mL of deionized water was carefully removed and replaced with 2 mL of fresh deionized water. The supernatant was filtered through a 0.2 µm filter before measuring phosphate (PO_4_) and calcium (Ca) ions.

To detect calcium ions in a previous study,^[^ ^40^ ^]^ the filtered supernatant was diluted twofold and measured using a Ca ion selective electrode (ISE, 9720BNWP, Thermo Fisher Scientific Inc.). Prior to the ISE measurements, the electrode was calibrated using a calcium ion standard solution (100 ppm, Orion 923206, Thermo Fisher Scientific Inc.) at three different concentrations (1, 10, and 100 ppm). Before conducting the experiments, the electrode was stored in a 100‐ppm standard solution to minimize interference. A calcium ionic strength adjuster (Orion 932011, Thermo Fisher Scientific Inc.) was used to adjust the ionic strength of each standard solution and sample according to the manufacturer's instructions. For PO_4_ ion detection in accordance with a previous study,^[^ ^41^ ^]^ the filtered supernatant was diluted tenfold and measured using an Orion AQUAfast II Chemistries Test Kit (reference AC2096, Thermo Fisher Scientific Inc.). The phosphate ion concentration in each sample was then measured with an Orion AQ3700 Colorimeter (Thermo Fisher Scientific Inc.). All experiments were performed at ambient temperatures (24 ± 1 °C).

### Cell Culture and Extract Preparation

For in vitro tests, MC3T3‐E1 pre‐osteoblastic cells (CRL‐2593, subclone 4, American Type Culture Collection, Manassas, VA, USA) were cultured in Alpha Minimum Essential Medium (α‐MEM, Hyclone Co., Logan, UT, USA) supplemented with 10% (v/v) fetal bovine serum (FBS, Gibco, Thermo Fisher Scientific, Waltham, MA, USA) and 1% (v/v) penicillin‐streptomycin (P/S, Hyclone) in an incubator with standard cell culture conditions of 37 °C and 5% CO₂. MC3T3‐E1 cells were used for cytocompatibility and quantitative polymerase chain reaction (qPCR) assays.

Human dental pulp stem cells (hDPSC, Lonza, Basel, Switzerland) were cultured in Dulbecco's Modified Eagle's Medium (DMEM, Welgene, Gyeongsan, Republic of Korea) supplemented with 10% (v/v) FBS (Gibco, Thermo Fisher Scientific) and 1% (v/v) P/S (Hyclone). These hDPSCs were cultured in an incubator (Thermo Fisher Scientific) under standard cell culture conditions of 37 °C and 5% CO₂. hDPSCs of passages 2–6 were used for all experiments in this study.

Specimens (diameter 10 mm, height 2 mm) were immersed in the growth medium at an extraction ratio of 3 cm^2^ mL^−1^ for 7 days at 37 °C.^[^ ^42^ ^]^ The extraction medium was then filtered through a 0.2 µm filter and diluted 100‐fold using this study. The odontogenic medium was prepared by supplementing the growth medium with 50 µg mL^−1^ ascorbic acid (Sigma–Aldrich) and 10 mm β‐glycerophosphate (Sigma–Aldrich). Similarly, the odontogenic extraction medium prepared that a 100‐fold diluted extraction medium was supplemented with 50 µg mL^−1^ ascorbic acid (Sigma–Aldrich) and 10 mm β‐glycerophosphate (Sigma–Aldrich).

### Cytocompatibility Assay

Each MC3T3‐E1 and hDPSCs was seeded into a 24‐well plate at a density of 2 × 10^4^ cells in the growth medium. After 24 h, the old medium was discarded and replaced with the respective extraction medium, and cells were cultured for either 1 day or 3 days. At each time point, cell viability was assessed using the LIVE/DEAD Viability/Cytotoxicity Kit (Thermo Fisher Scientific) according to the manufacturer's instructions. A fluorescence microscope (EVOS FL, Thermo Fisher Scientific Inc.) was used to observe cells, with live cells stained green and dead cell‐stained red.

Additionally, a water‐soluble tetrazolium‐1 (WST‐1, EZ‐Cytox, DoGenBio, Republic of Korea) assay was performed to analyze cytotoxicity. After culturing cells for 1 day or 3 days in the extraction medium, 10% WST‐1 solution in the fresh growth medium was added and incubated for 1 h at 37 °C. The optical density was measured at 450 nm using a microplate reader (BioTek, Winooski, VT, USA). The growth medium without extraction served as a mock control.

### Osteogenic/Odontogenic Gene Expression

MC3T3‐E1 cells were seeded into a 6‐well plate at a density of 1 × 10⁵ cells per well. After 24 h of cell adhesion, the old medium was discarded and replaced with the extraction medium for 7 days. On the other hand, hDPSC were seeded into a 12‐well plate at a density of 1.5 × 10⁵ cells per well. After 24 h of cell attachment, the old medium was discarded and replaced with the extracted odontogenic medium as mentioned above for culturing of 7 and 14 days. At each time point, total RNA was extracted using Trizol reagent (Qiazol, Qiagen, Hilden, Germany) and quantified using a Nanodrop 2000 spectrophotometer (Thermo Fisher Scientific). cDNA synthesis was performed using a PrimeScript RT reagent kit (Takara, Tokyo, Japan) according to the manufacturer's instructions. qPCR assay was conducted using a Real‐Time PCR system with SYBR Premix Ex Taq II (Tli RNaseH Plus), and ROX Plus (Takara). Target gene primers were purchased from Bioneer (Daejeon, Republic of Korea) (Table S2, Supporting Information). PCR conditions were: 95 °C for 30 s, followed by 40 cycles at 95 °C for 5 s and 60 °C for 30 s. Gene expression was quantified using the 2^−ΔΔCT^ method and normalized to glyceraldehyde‐3‐phosphate dehydrogenase (GAPDH).

### Measurement of IL‐6 Cytokine from hDPSCs

Cytokine concentration in the supernatant was measured using enzyme‐linked immunosorbent assay (ELISA) kits. hDPSCs were seeded into a 24‐well plate at a density of 1.5 × 10⁵ cells per well. The old medium was discarded and replaced with the extracted odontogenic medium, which was refreshed at 3‐day intervals over the indicated period. After 14 days of culture, the level of interleukin‐6 (IL‐6) was analyzed using an ELISA kit (OptEIA Set, BD Biosciences, San Jose, CA) according to the manufacturer's instructions. For the assay, 100 µL of each supernatant was combined with the assay buffer. The absorbance was then measured at 450 nm using an ELISA plate reader (Synergy 2, Bio‐Tek).

### Western Blot Assay

To detect odontogenic differentiation at the protein level, western blot analysis was performed. hDPSCs were cultured by growth medium in 100 mm culture dishes (SPL Life Science, Seoul, Korea) until they reached 80% confluence. The growth medium was then switched to odontogenic medium or extracted odontogenic medium and cells were cultured for 7 and 14 days. At each time point, cells were lysed using a buffer containing 50 mm Tris‐HCl (pH 8.0), 5 mm EDTA, 1% NP‐40, 150 mm NaCl, 1 mM aprotinin, 1 mm pepstatin, and 100 nm leupeptin. Total protein was quantified using the Bradford dye‐binding procedure (Bio‐Rad, Hercules, CA, USA). An aliquot of 20 µg of protein from each sample was separated on 8% to 10% sodium dodecyl sulfate‐polyacrylamide gel under denaturing conditions and transferred to a Hybond‐P membrane (Amersham, Arlington, IL, USA) using a Mini‐Protean II system (Bio‐Rad). After blocking with 5% skim milk in phosphate‐buffered saline (PBS), membranes were incubated with primary antibodies against RUNX2 (#12556, Cell Signaling Technology, Beverly, MA, USA), DSPP (sc‐73632, Santa Cruz Biotechnology, CA, USA), DMP1 (sc‐73633, Santa Cruz Biotechnology, CA, USA), ICAM‐1 (sc‐8439, Santa Cruz Biotechnology, CA, USA), and VCAM‐1 (sc‐13160, Santa Cruz Biotechnology, CA, USA) at a 1:1000 dilution in 3% bovine serum albumin overnight at 4 °C. After washing with PBS containing 0.1% Tween‐20 (Sigma–Aldrich), membranes were incubated with secondary antibodies—either anti‐mouse or anti‐rabbit immunoglobulin (IgG) conjugated to horseradish peroxidase—at a 1:3000 dilution in PBS for 1 h at 25 °C. Protein bands were visualized using chemiluminescent detection (Amersham Pharmacia Biotech, London, UK) and detected with a LAS‐4000 scanner (LAS‐4000, Fuji Film, Tokyo, Japan). The effect of PIMC on hard tissue regeneration in dental pulp in vivo was then determined.

### Dog Pulpotomy In Vivo Model for Dental Hard Tissue Regeneration

For the in vivo assessment of dental hard tissue regeneration, a dog pulpotomy model was used as described in previous studies.^[^ ^43^ ^]^ All experiments were performed in compliance with the ARRIVE guidelines. They were approved by the Institutional Animal Care and Use Committee of Yonsei University Health System, Seoul, Republic of Korea (Approval No. 2021‐0130). All experiments were carried out according to relevant guidelines and regulations. Two mongrel dogs at 10–14 months of age with weights of 25–30 kg were included and their teeth were divided into two groups: 1) a control group treated with the MC; and 2) an experimental group treated with 5PIMC. A split‐mouth design was adopted,^[^ ^44^ ^]^ with one tooth and the contra‐lateral tooth of an animal assigned to control and experimental groups, respectively.

After general anesthesia, pulpotomies were aseptically performed on canines and premolars. After exposing pulp tissues above root canal orifices using a diamond bur, MC or 5PIMC was mixed and placed directly on pulp tissues of ≈3 mm thickness according to treatment groups. Afterward, the tooth cavity was sealed with a dental composite resin (Tetric N‐Flow, Ivoclar Vivadent, Schaan, Liechtenstein) to minimize microleakage and provide a robust barrier against external salivary contaminants, ensuring optimal protection and longevity of the restoration. Periapical radiographs were taken before and after the operation to confirm the surgical procedure. Animals were prescribed 30 mg kg^−1^ cefazolin sodium (Cefazolin; Chongkundang, Seoul, Republic of Korea), ketorolac tromethamine (Keromin; Hana Pharm, Seoul, Republic of Korea), and 0.2 mg kg^−1^ meloxicam (Mobic; Boehringer Ingelheim, Ingelheim, Greece) for a week. At 8 weeks after the operation, animals were sacrificed using an overdose of potassium under general anesthesia. After taking periapical radiographs, all teeth were extracted with forceps (Physics Forceps, GoldenDent, Detroit, MI, USA) and fixed in 10% buffered paraformaldehyde. To unitize specimens for analysis, two‐rooted teeth were hemisected into two individual single roots before extraction.

### Micro‐Computed Tomography Scanning and Analysis

Tooth specimens were examined using a µ‐CT imaging system (Quantum GX, PerkinElmer, Hopkinton, MA, USA) at an isotropic resolution of 40 µm. Images of each sample were evaluated according to hard tissue in the pulp space using 3D Slicer 4.11.0 software (https://www.slicer.org/). The amount of hard tissue formation in the pulp space and the ratio of hard tissue to soft tissue were calculated and compared between the experimental group and the control group.

### Histological Staining

To perform histology analysis, tooth specimens were decalcified in 10% ethylenediaminetetraacetic acid (EDTA) solution (pH 7.4) at 4 °C for 8 weeks and then embedded in paraffin. Prepared paraffin blocks were cut at a thickness of 3 µm into sagittal sections containing the pulp tissue. Sections were stained using hematoxylin and eosin dye. In addition, Masson's trichrome staining was performed using a trichrome staining kit (SSK5005‐250, BBC Biochemical, WA, USA) according to the manufacturer's instructions. These sections were then examined using a light microscope (BX50, Olympus, Tokyo, Japan) and an auto slide scanner (TW‐SM01, TaeWoong Medical Co.Ltd., Goyang, Republic of Korea).

### Statistical Analysis

SPSS 26 software (IBM, Armonk, NY, USA) was used for all statistical analyses. All quantitative results are presented as mean ± standard deviation (SD) after at least three independent experiments. Data were assessed by one‐way analysis of variance (ANOVA) followed by Tukey's post‐hoc test. Comparisons of qPCR data between two groups were performed using a *t*‐test and µ‐CT analysis data between two groups were performed using Mann–Whitney U test. The significance level was set at *p* < 0.05.

## Conflict of Interest

The authors declare no conflict of interest.

## Ethics Approval Statement

All animal experiments were performed in compliance with the ARRIVE guidelines. They were approved by the Yonsei University Health System Institutional Animal Care and Use Committee, Seoul, Republic of Korea (Approval No. 2021‐0130). All experiments were carried out according to relevant guidelines and regulations.

## Supporting information



Supporting Information

## Data Availability

The data that support the findings of this study are available from the corresponding author upon reasonable request.
