# Supplementary material for: Integrating Phosphate Enhances Biomineralization Effect of Methacrylate Cement in Vital Pulp Treatment with Improved Human Dental Pulp Stem Cells Stimulation
Source: Adv Healthc Mater. 2024 Oct 4;13(31):2402397. doi: 10.1002/adhm.202402397 (PMC11650430; doi:10.1002/adhm.202402397)
Supplement: Supplementary file 1 — Supporting Information [file ADHM-13-0-s001.pdf]

ADVANCED HEALTHCARE MATERIALS

Supporting Information

for *Adv. Healthcare Mater.*, DOI 10.1002/adhm.202402397

Integrating Phosphate Enhances Biomineralization Effect of Methacrylate Cement in Vital Pulp Treatment with Improved Human Dental Pulp Stem Cells Stimulation

Jeong-Hyun Ryu, Utkarsh Mangal, Jae-Sung Kwon, Ji-Young Seo, Seong-Yun Byun, Young-Hee Lee, Sungil Jang, Geelsu Hwang, Hyemin Ku, Yooseok Shin, Dohyun Kim and Sung-Hwan Choi**

Supporting Information

Integrating Phosphate Enhances Biomineralization Effect of Methacrylate Cement in Vital Pulp Treatment with Improved Human Dental Pulp Stem Cells Stimulation

Jeong-Hyun Ryu[1#], Utkarsh Mangal[2#], Jae-Sung Kwon[2,3,#], Ji-Yeong Seo[1], Seong-Yun Byun[3], Young-Hee Lee[4], Sungil Jang[4], Kee-Joon Lee[1], Jung-Yul Cha[1], Geelsu Hwang[5,6], Hyemin Ku[7], Yooseok Shin[7], Dohyun Kim[7,], Sung-Hwan Choi[1,*]*

[1] Department of Orthodontics, Institute of Craniofacial Deformity, Yonsei University College of Dentistry, 50-1 Yonsei-ro, Seodaemun-gu, Seoul, Republic of Korea.

[2] BK21 FOUR Project, Yonsei University College of Dentistry, 50-1 Yonsei-ro, Seodaemun-gu, Seoul, Republic of Korea.

[3]Department and Research Institute of Dental Biomaterials and Bioengineering, Yonsei University College of Dentistry, 50-1 Yonsei-ro, Seodaemun-gu, Seoul, Republic of Korea.

[4]Department of Oral Biochemistry, Institute of Oral Bioscience, School of Dentistry, Jeonbuk National University, Jeonju-si 54907, Republic of Korea.

[5]Department of Preventive and Restorative Sciences, School of Dental Medicine, University of Pennsylvania, Philadelphia, PA, 19104, United States

[6]Center for Innovation & Precision Dentistry, School of Dental Medicine, School of Engineering and Applied Sciences, University of Pennsylvania, Philadelphia, PA, United States

[7]Department of Conservative Dentistry and Oral Science Research Center, Yonsei University College of Dentistry

[#]These authors contributed equally

*Corresponding authors:

Dohyun Kim (@yuhs.ac); Sung-Hwan Choi (selfexam@yuhs.ac).

Supplementary Tables

Table S1. The composition of phosphate-based glass (PBG) used in this study

Code	P_2O_5	CaO	Na_2O
**PBG**	**50 mol%**	**30 mol%**	**20 mol%**

P_2O_5, phosphorus pentoxide; CaO, calcium oxide; Na_2O, sodium oxide

Table S2. Primer sequences used for gene expression

Type	Molecules	Primer sequence (5'→3')	Product size (bp)	Accession No.
**Human**	RUNX2	Forward: TTCGCCTCACAAACAACCAC<br>Reverse: GCTTGCAGCCTTAAATGACTC	145	NM_001024630.4
	OCN	Forward: CACCGAGACACCATGAGAGC<br>Reverse: CTGCTTGGACACAAAGGCTGC	132	NM_199173.6
	DSPP	Forward: GGAAGAGCCAAGATAAGGGAAT<br>Reverse: GTCTTGACATTGCCTTTGCCC	142	NM_014208.3
	DMP1	Forward: CCTGAGGATGAGAACAGCTCCA<br>Reverse: GATCTGCTGCTGTCTTGAGAGTCAC	122	NM_004407.4
	GAPDH	Forward: TCGGAGTCAACGGATTTGGT<br>Reverse: TTCCCGTTCTCAGCCTTGAC	181	NM_002046.7
**Mouse**	RUNX2	Forward: GGGAACCAAGAAGGCACAGA<br>Reverse: ACTTGGTGCAGAGTTCAGGG	152	NM_001271627.1
	OSX	Forward: GTCCTCTCTGCTTGAGGAAGAA<br>Reverse: TCTTTGTGCCTCCTTTCCCC	131	NM_130458.4
	SPARC	Forward: GGCGAGTTTGAGAAGGTATGC<br>Reverse: TGGTCCGATGTAGTCCAGGT	129	NM_001290817.1
	OPN	Forward: GAGGAAACCAGCCAAGGACTAA<br>Reverse: TCTGGGTGCAGGCTGTAAA	140	NM_009263.3
	OCN	Forward: TTGGCCCAGACCTAGCAGA<br>Reverse: CTGGGCTTGGCATCTGTGA	100	NM_007541.3
	BSP	Forward: CGGTTTCCAGTCCAGGGAGG<br>Reverse: CGAGAGTGTGGAAAGTGTGGA	174	NM_008318.3
	GAPDH	Forward: CCCACTCTTCCACCTTCGATG<br>Reverse: CGAGTTGGGATAGGGCCTCT	201	NM_001289726.1

RUNX2, runt-related transcription factor 2; OCN, osteocalcin; DSPP, dentin sialophosphoprotein; DMP1, dentin matrix protein 1; OSX, osterix; SPARC, secreted protein acidic and rich in cysteine; OPN, osteopontin; BSP, bone sialoprotein; GAPDH, glyceraldehyde-3-phosphate dehydrogenase.

Table S3. The compressive strength and degradation rate immersed in the deionized water for 7 days from PIMC groups

Code	Before immersion (MPa)	After immersion (MPa)	Degradation rate (%)
MC	84.61 ± 6.16^{a}	84.32 ± 8.29^{a}	0.128 ± 0.031^{a}
5PIMC	84.38 ± 6.07^{a}	79.20 ± 7.33^{a}	0.586 ± 0.080^{a}
10PIMC	85.33 ± 5.50^{a}	72.91 ± 4.36^{a}	1.190 ± 0.352^{a}
20PIMC	86.60 ± 7.75^{a}	50.06 ± 11.03^{b}	4.868 ± 1.489^{b}
p-value	9.3×10^{-3}	2.3×10^{-6}	1.3×10^{-7}

MC; methacrylate-based cement, PIMC; phosphate-based glass integrated MC.

The data exhibited the mean and standard deviation. All data were performed by one-way analysis of variance (ANOVA) with Tukey's post hoc test with significance at a $p < 0.05$. The different small letters indicate significant differences between groups at the same time point.

Table S4. The ionic concentration (mM) of calcium release pattern from the PIMC groups

	MC	5PIMC	10PIMC	20PIMC	*p*-value
2 h	0.000^{a}	0.085 ± 0.023^{b}	0.084 ± 0.015^{b}	0.214 ± 0.035^{c}	1.9×10^{-6}
4 h	0.000^{a}	0.166 ± 0.028^{b}	0.206 ± 0.034^{b}	0.527 ± 0.099^{c}	1.6×10^{-6}
8 h	0.000^{a}	0.295 ± 0.029^{b}	0.391 ± 0.056^{b}	0.959 ± 0.174^{c}	1.0×10^{-6}
24 h	0.000^{a}	0.536 ± 0.035^{b}	0.758 ± 0.100^{b}	1.787 ± 0.313^{c}	7.1×10^{-6}
48 h	0.000^{a}	0.946 ± 0.063^{b}	1.403 ± 0.185^{b}	3.323 ± 0.593^{c}	7.8×10^{-6}
72 h	0.000^{a}	1.414 ± 0.084^{b}	2.200 ± 0.263^{b}	5.136 ± 0.938^{c}	8.5×10^{-6}
168 h	0.000^{a}	2.239 ± 0.079^{b}	3.665 ± 0.244^{b}	8.642 ± 1.623^{c}	8.1×10^{-6}

MC; methacrylate-based cement, PIMC; phosphate-based glass integrated MC.

The data displayed the mean and standard deviation. All data were performed by one-way analysis of variance (ANOVA) with Tukey's post hoc test with significance at a $p < 0.05$. The different small letters indicate significant differences between groups at the same time point.

Table S5. The ionic concentration (mM) of calcium release pattern from the PIMC group.

	PMMA	5PMMA	10PMMA	20PMMA	p-value
2 h	0.000^{a}	0.129 ± 0.056^{b}	0.204 ± 0.049^{b}	0.420 ± 0.116^{c}	5.0×10^{-5}
4 h	0.000^{a}	0.301 ± 0.049^{b}	0.506 ± 0.049^{c}	0.882 ± 0.067^{d}	1.2×10^{-7}
8 h	0.000^{a}	0.689 ± 0.075^{b}	0.915 ± 0.104^{b}	1.431 ± 0.166^{c}	1.3×10^{-6}
24 h	0.000^{a}	1.044 ± 0.075^{b}	1.442 ± 0.113^{b}	2.088 ± 0.293^{c}	1.8×10^{-6}
48 h	0.000^{a}	1.442 ± 0.130^{b}	2.034 ± 0.085^{c}	3.078 ± 0.416^{d}	1.1×10^{-6}
72 h	0.000^{a}	2.012 ± 0.122^{b}	2.733 ± 0.067^{c}	4.229 ± 0.434^{d}	1.2×10^{-7}
168 h	0.000^{a}	2.723 ± 0.149^{b}	3.648 ± 0.085^{c}	5.994 ± 0.476^{d}	1.7×10^{-8}

MC; methacrylate-based cement, PIMC; phosphate-based glass integrated MC.

The data displayed the mean and standard deviation. All data were performed by one-way analysis of variance (ANOVA) with Tukey's post hoc test with significance at a $p < 0.05$. The different small letter was a significant difference.

Table S6. μ-CT analysis results from 5PIMC against MC.

Code	Newly formed HT volume (mm^3)	HT volume per pulp space (%)
MC	0.036 ± 0.039^a	0.180 ± 0.117^a
5PIMC	0.231 ± 0.252^b	0.699 ± 0.211^b

MC; methacrylate-based cement, PIMC; phosphate-based glass integrated MC. HT; hard tissue. Different lowercase letters indicate statistically significant difference observed through Mann-Whitney U test at significance level of $p < 0.05$.

Supplementary Figures

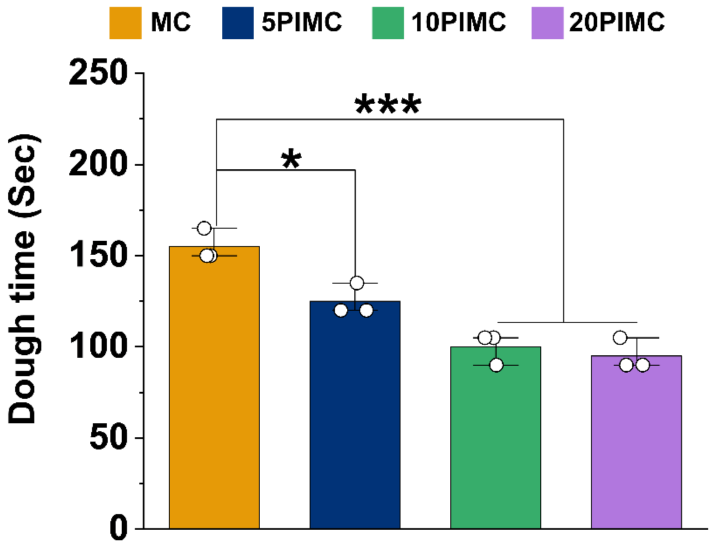

Figure S1. Measurement of the dough time to the PIMC groups. The dough time for 5PIMC, 10PIMC, and 20PIMC were reduced by *ca.* 19.4%, 35.5%, and 38.7% against MC, respectively ($n = 3$; * $p < 0.05$, *** $p < 0.001$). MC; methacrylate-based cement, PIMC; phosphate-based glass integrated MC.

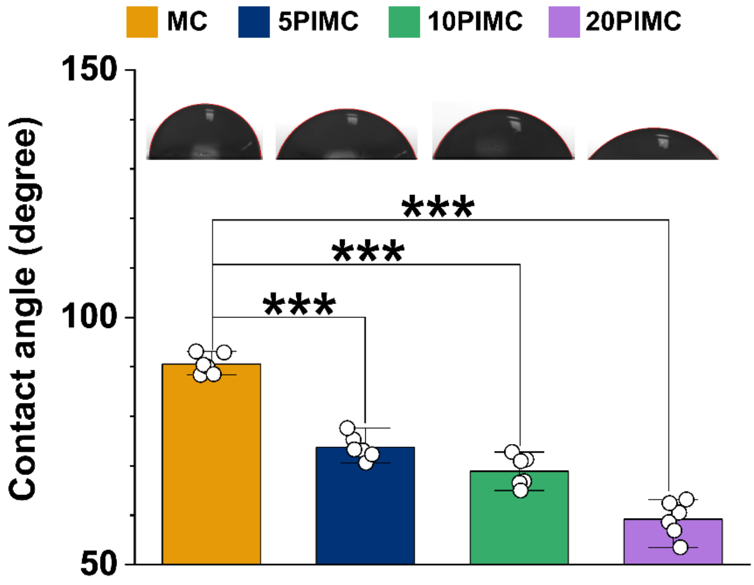

Figure S2. Measurement of surface wettability to the PIMC group. The surface wettability for 5PIMC, 10PIMC, and 20PIMC were reduced by ca. 18.6%, 23.9%, and 34.6% against MC, respectively ($n = 6$; *** $p < 0.001$). MC; methacrylate-based cement, PIMC; phosphate-based glass integrated MC.

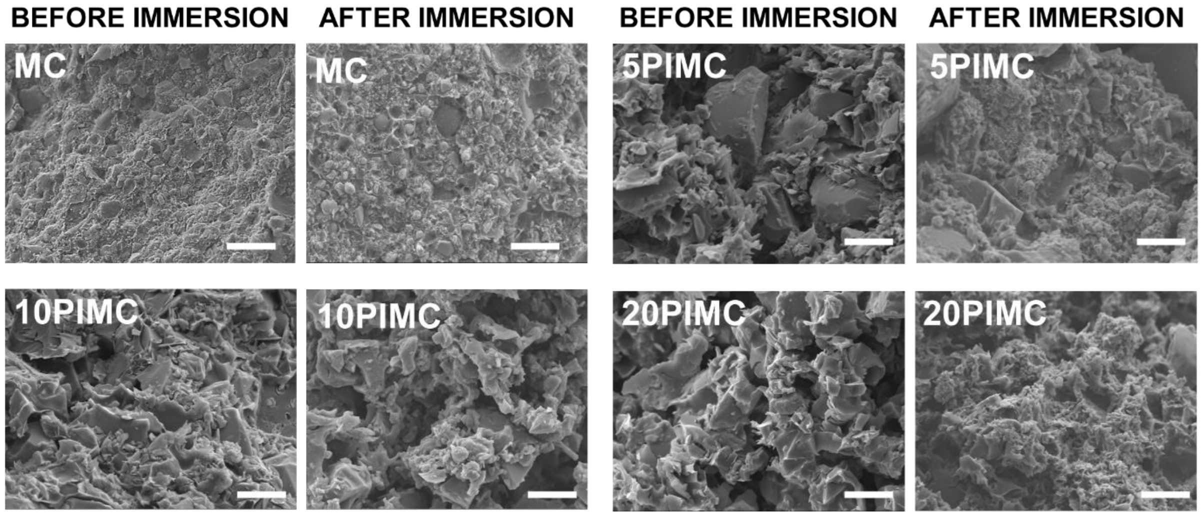

Figure S3. Extended data from Figure 2 contrasting changes in surface morphology after immersion of the MC and PIMC samples. MC, methacrylate-based cement ; PIMC, PBG-integrated MC; PBG, phosphate-based glass

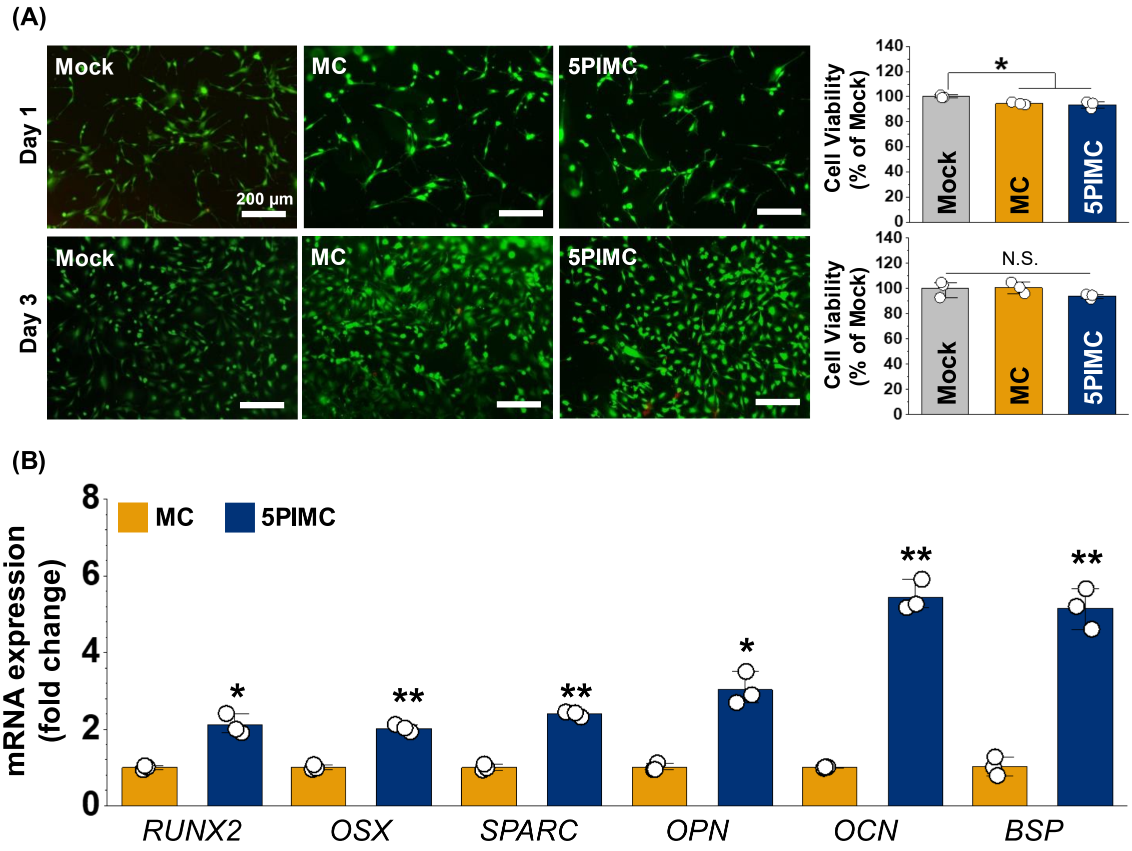

Figure S4. Murine pre-osteoblast (MC3T3-E1) cell assay. (A) Fluorescent staining microscopic images and cell viability results for mock, methacrylate cement (MC), and 5% phosphate-integrated methacrylate cement (5PIMC) at days 1 and 3. Relative gene expression of RUNX2, OSX, SPARC, OPN, OCN, and BSP in early osteogenic activity at day 7 between MC and 5PIMC (n = 3; *$p < 0.05$, **$p < 0.01$, ***$p < 0.001$).

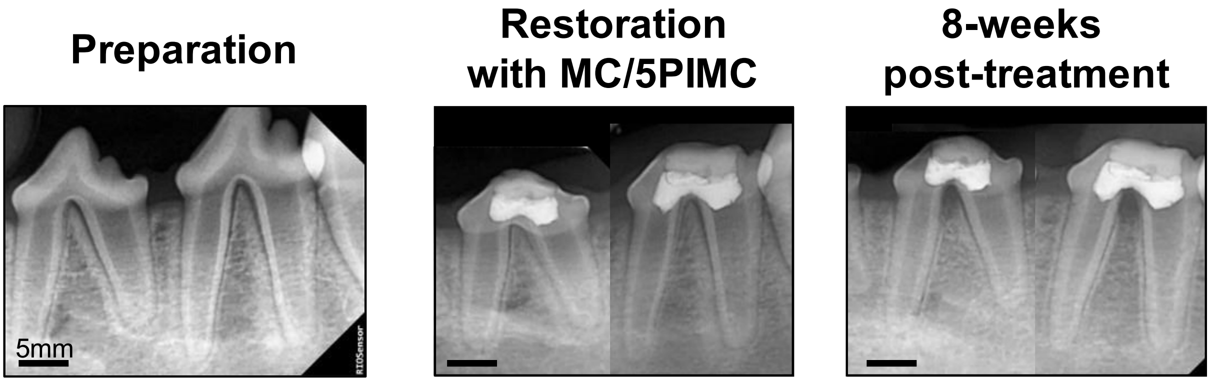

Figure S5. Radiographs during in vivo vital pulp therapy procedures.
